# The Lifecycle of Electronic Health Record Data in HIV-Related Big Data Studies: Qualitative Study of Bias Instances and Potential Opportunities for Minimization

**DOI:** 10.2196/71388

**Published:** 2025-08-07

**Authors:** Arielle N'Diaye, Shan Qiao, Camryn Garrett, George Khushf, Jiajia Zhang, Xiaoming Li, Bankole Olatosi

**Affiliations:** 1 Department of Health Promotion, Education, and Behavior, Arnold School of Public Health, University of South Carolina, Discovery 410, 915 Greene Street, Columbia, SC, 29208, United States, 1 803-777-6844; 2 Department of Philosophy, College of Arts and Sciences, University of South Carolina, Columbia, SC, United States; 3 Department of Epidemiology and Biostatistics, Arnold School of Public Health, University of South Carolina, Columbia, SC, United States; 4 Department of Health Services, Policy, and Management, Arnold School of Public Health, University of South Carolina, Columbia, SC, United States

**Keywords:** electronic health records, HIV, big data, bias, deep south, social determinants of health

## Abstract

**Background:**

Electronic health record (EHR) data are widely used in public health research, including in HIV-related studies, but are limited by potential bias due to incomplete and inaccurate information, lack of generalizability, and lack of representativeness.

**Objective:**

This study explores how workflow processes among HIV health care providers (HCPs), data scientists, and state health department professionals may potentially introduce or minimize bias within EHR data.

**Methods:**

One focus group with 3 health department professionals working in HIV surveillance and 16 in-depth interviews (ie, 5 people with HIV, 5 HCPs, 5 data scientists, and 1 health department professional providing retention-in-care services) were conducted with participants purposively sampled in South Carolina from August 2023 to April 2024. All interviews were transcribed verbatim and analyzed using a constructivist grounded theory approach, where transcripts were first coded and then focused, axial, and theoretically coded.

**Results:**

The EHR data lifecycle originates with people with HIV and HCPs in the clinical setting. Data scientists then curate EHR data and health department professionals manage and use the data for surveillance and policy decision-making. Throughout this lifecycle, the three primary stakeholders (ie, HCPs, data scientists, and health department professionals) identified challenges with EHR processes and provided their recommendations and accommodations in addressing the related challenges. HCPs reported the influence of socio-structural biases on their inquiry, interpretation, and documentation of social determinants of health (SDOH) information of people living with HIV, the influence of which is proposed to be mitigated through people living with HIV access to their EHRs. Data scientists identified limited data availability and representativeness as biasing the data they manage. Health department professionals face challenges with delayed and incomplete data, which may be addressed statistically but require consideration of the data’s limitations. Overall, bias within the EHR data lifecycle persists because workflows are not intentionally structured to minimize bias and there is a diffusion of responsibility for data quality between the various stakeholders.

**Conclusions:**

From the perspective of various stakeholders, this study describes the EHR data lifecycle and its associated challenges as well as stakeholders’ accommodations and recommendations for mitigating and eliminating bias in EHR data. Based upon these findings, studies reliant on EHR data should adequately consider its challenges and limitations. Throughout the EHR data lifecycle, bias could be reduced through an inclusive, supportive health care environment, people living with HIV verification of SDOH information, the customization of data collection systems, and EHR data inspection for completeness, accuracy, and timeliness. Future research is needed to further identify instances where bias is introduced and how it can best be mitigated and eliminated across the EHR data lifecycle. Systematic changes are necessary to reduce instances of bias between data workflows and stakeholders.

## Introduction

Electronic health record (EHR) data are widely used in public health research. In HIV research, EHR data are used for disease surveillance, to understand treatment uptake, to assess the efficacy of treatment regimens, and to examine health outcome disparities [1-3]. As data sources, EHR data are an attractive option because of their cost-effectiveness, the high volume of population-level data they yield, and their suitability for multidimensional analyses [1,2,4]. However, existing literature notes that EHR, as a data source, is limited by potential bias from incomplete and inaccurate information, lack of generalizability, and underrepresentation [4]. For example, EHR data are subject to context-specific biases, such as the disproportionate likelihood that underinsured and uninsured individuals are excluded from EHR systems in states that did not expand Medicaid (eg, South Carolina, Mississippi, Texas, Alabama, and Florida) [5-7]. Many scholars attribute these limitations to the origin and design of EHR for medical billing, scheduling, and clinical record keeping, rather than for public health research [4]. Scholars also highlight the vulnerability of EHR data to social biases, such as discrepancies in if and how information is entered into EHR systems, as a limitation [8,9]. The existing literature notes that inaccuracies are prevalent across all levels of EHR data (eg, within private health care systems, public health care systems, state surveillance systems, and federal surveillance systems) [10].

EHR data used in HIV research have been found to be vulnerable to biases introduced during data collection, including HIV-related stigma, discrimination, and adversity in relation to the social determinants of health [3]. Within health care settings, more broadly, EHR data biases are observed as stemming from institutional policies, training practices, and health care provider (HCP) biases, which in turn affect the accuracy, completeness, and representativeness of EHR data [8,9]. For example, among people living with HIV, laboratory testing and EHR-based HIV surveillance are at risk of inaccuracies and underrepresentation because they can only include those who know their HIV status and who are engaged in care. Furthermore, the implicit biases of HCPs, when providing HIV care, influence the inquiry, interpretation, and EHR documentation of sexual history of people living with HIV, which has been found to exacerbate patient challenges with accessing HIV prevention resources (eg, pre-exposure prophylaxis and HIV counseling) [11-13]. The effects of HCPs’ implicit biases disproportionately affect lesbian, gay, bisexual, transgender, and queer (LGBTQ) individuals, women, and racial minorities [12,13]. Although implicit biases among HCPs toward LGBTQ individuals occur throughout the United States, the existing literature observes these experiences as being particularly prevalent within the Southern United States [14-16]. The current literature offers a variety of techniques to address bias derived from inaccurate, incomplete, and under-representative data, including the imputation of surrogate measures to address missing information, conducting validation studies with external datasets to determine the representativeness of EHR data, performing sensitivity analyses to identify misclassified information, using causal diagrams to support causal inferences, and using record audits to assess data quality [17-20].

In addition to retroactively coping with challenges to EHR data quality (eg, by using advanced data science analytics), there persists the need to proactively describe the EHR data lifecycle and identify where bias may be introduced and mitigated, from the perspectives of various stakeholders (eg, people living with HIV, HCPs, data scientists, and health department professionals). The literature has called for further research into the EHR data lifecycle in response to findings of a lack of standardized workflows in the extraction, preparation, and use of EHR data in research [21]. In addition, there is a lack of studies regarding the lived experiences of diverse, key stakeholders who are engaged in different stages of the EHR data lifecycle, including people living with HIV, HCPs, and data scientists [22]. Therefore, this study aims to fill the abovementioned knowledge gaps by describing the EHR data lifecycle through the individual workflows of key stakeholders and by identifying instances where bias is introduced and potential opportunities for mitigation within the collection, curation, management, and usage of EHR data. To our knowledge, this study is the first of its kind to explore instances of bias introduction and mitigation across the EHR data lifecycle, within the data collection, curation, management, and usage workflows of HIV clinics, data scientists, and health department professionals [11-13]. The present work was guided by the following overarching research questions: (1) How is bias introduced within health care and public health research workflows and across the EHR data lifecycle? (2) How might these workflows be structured to minimize opportunities for bias in EHR big data?

## Methods

### Theoretical Approach and Research Paradigm

This study used a constructivist grounded theory approach, as outlined by Charmaz [23], that holds the worldview that researchers play an active role in defining and shaping meanings derived from the data [24]. This theoretical approach was deemed best suited to answer the research questions, because of the emphasis on dynamic collaboration throughout the data analysis process [25]. Throughout the study, the research team engaged in researcher reflexivity and reported personal characteristics, relevant to this study, including their academic training (eg, public health and sociology), positionality as public health researchers, and experience conducting qualitative research.

### Sampling Strategy and Recruitment

Participants were purposively sampled to represent the perspectives of people living with HIV, HCPs, data scientists (ie, who provide EHR datasets to public health researchers), and health department professionals (ie, who work with EHR datasets). All recruited participants were at least 18 years of age. People living with HIV were eligible for inclusion if they were living in South Carolina. HCPs recruited for this study were those who provide HIV care in South Carolina and use EHR systems. Data scientists were eligible for inclusion if they were professional data curators, data management experts, or data repository administrators. Finally, public health department professionals were recruited if they were employed by state agencies; engaged in EHR data management and usage; and oversaw HIV surveillance, retention-in-care, or South Carolina’s public data repositories.

All participants were recruited through referrals from a local research partner network, which consists of stakeholders from AIDS service organizations, community-based organizations, academia, state public health agencies, people living with HIV, and state policymakers. After contacting a member of the research team, participants were provided with an invitation to participate that outlined study activities and the voluntary nature of participation. This recruitment strategy yielded 16 participants (ie, 5 people living with HIV, 5 HCPs, 5 data scientists, and 1 health department professional providing retention-in-care services). This recruitment strategy also yielded a focus group (n=1) with three health department professionals working in HIV surveillance. Study participants were recruited from August 2023 to April 2024 and received a US $50 gift card for their participation in this study.

### Data Collection

Individual in-depth interviews (n=16) were conducted by research team members using semistructured interview guides. The interview guides were tailored to the contexts and responsibilities of the key stakeholders. All stakeholders were asked to describe challenges and recommendations or accommodations to their identified challenges, as part of the interview. People living with HIV were asked to describe their interactions with HCPs, their comfort in answering sensitive questions asked by their HCPs, and any concerns related to differential or unfair treatment and outcomes, as related to their health care. HCPs were asked to identify information that is necessary for patient care that might not be captured through EHR data, to describe the limitations of EHRs, and to share suggestions for improving EHR data and systems. Data scientists, who serve as data brokers, were asked to detail the process of data curation, how they respond to and prioritize data inquiries submitted to their agency, as well as to describe issues related to data quality and their impact on analyses and interpretations. Health department professionals were asked to detail their process of data curation, for suggestions regarding data that is needed but unrecorded by current EHR systems, if certain populations are excluded from EHR data, and how EHR data are used to inform policy decision-making and resource allocation. All questions of the corresponding interview guide were asked of each participant, but allowed flexibility for the interviewer to probe for additional detail and clarification as needed.

A total of 6 interviews were conducted over videoconference platforms (eg, Zoom [Zoom Video Communications] and Microsoft Teams) and 10 interviews were conducted in person. Interviews were conducted using both mediums because the existing literature finds both online and in-person interview formats to be comparable [26]. Participants interveiwed online were advised to be in a location where they were comfortable and able to have a private conversation. In-person interviews were conducted in a private room at a location that was convenient for the participant. Interviews ranged from 45 to 60 minutes in length and were audio recorded. The focus group (n=1) was conducted in person, in a private office at the participants’ workplace. The focus group lasted for 73 minutes and used the same semistructured interview guide as the corresponding, individual in-depth interview.

### Data Analysis

Transcripts were first transcribed using Otter.ai (version 3.43.2-240212-89103881, Otter.ai, Inc), a transcription service that maintains a variety of privacy and security measures (ie, SOC 2 Type 2, General Data Protection Regulation [GDPR], California Consumer Privacy Act) [27]. Transcripts were then reviewed, line-by-line, by the research team to ensure accuracy and verbatim transcription. Any potentially identifying information (eg, names of partners, family members, schools, and workplaces) was removed during transcript cleaning. All data were managed and analyzed in Microsoft Excel (Version 16.94). Individual interviews were the unit of analysis.

Each full transcript was initially coded line-by-line. These codes were then focused, axial, and theoretically coded [23]. Throughout the analytical process, constant comparison was used to identify emergent trends within the data. The initial analysis was conducted by one member of the research team (AN). Codes and latent level categorizations were reviewed and discussed with another author (CG). An analytical matrix of study findings was used to establish consensus among all authors (AN, SQ, CG, KH, BO, ZJ, and XL). Author AN maintained a reflexive journal throughout the data analysis process, which was discussed when interpreting study results to limit the introduction of potential researcher biases. An audit trail was used throughout data collection and analysis to increase the credibility of the study’s results. This study was reported following the Standards for Reporting Qualitative Research (SRQR) checklist [28] (Checklist 1).

### Ethical Considerations

This study received institutional review board approval from The University of South Carolina Office of Research Compliance (application Pro00122501) and was conducted in accordance with the tenets of the Declaration of Helsinki [29]. All participant audio recordings and original transcripts were stored in a dual authentication, password-protected cloud drive and were accessible only to members of the research team. Audio recordings were deleted after transcribing. However, the transcripts, which serve as the dataset of this study, will be readily available for the United States and global research communities in accordance with National Institutes of Health (NIH) policies. At the beginning of each interview, participants received the invitation to participate and gave their verbal consent, as, due to the nature of the study, they were not required to give written consent. Furthermore, at the start of each interview, participants were assigned an ID that corresponded to their respective transcripts to protect participant confidentiality.

## Results

### Overview

**Figure 1.**
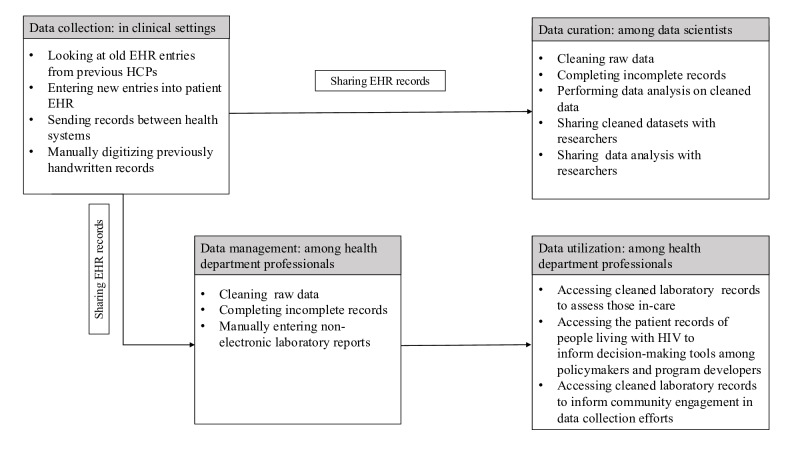
The electronic health record data lifecycle through stakeholder workflows. EHR: electronic health record; HCP: health care provider.

Throughout the EHR data lifecycle, a variety of stakeholders are responsible for the data collection, curation, management, and usage (Figure 1). As depicted in Figure 1, the EHR data lifecycle begins in the clinical setting where data are collected from patients by HCPs. Within our system, the information recorded in a patient’s electronic health record is then shared and accessible to both data scientists and local health department professionals. Data scientists then curate the data (eg, data cleaning, data imputation, and data analysis) for distribution to researchers. Health department professionals also curate and manage EHR data for usage in surveillance and policy decision-making.

The findings of the present study describe instances where bias is introduced over the EHR data lifecycle, ways in which stakeholders work to mitigate biases, and their recommendations for structural interventions to mitigate bias (Table 1). Table 1 depicts the perspectives of three key stakeholders who are associated with EHR data collection, curation, or management and usage. The perspectives captured include stakeholders’ primary challenges with EHR data processes as well as their recommendations and accommodations to address the aforementioned challenges. HCPs described challenges to data collection in clinical settings, such as the influence of socio-structural biases on their inquiry, interpretation, and documentation of social determinants of health (SDOH) information of people living with HIV. Data scientists, within their data curation workflow, illustrated the implications of limited data availability and representativeness biasing the data they manage. Health department professionals described the primary challenges and implications associated with EHR data management and usage, such as the influence of delayed and incomplete data on research products and tools for public health decision-making. Importantly, limitations to reduce bias within participants’ workflows persisted because (1) workflows are not intentionally structured to minimize bias and (2) the EHR data lifecycle is comprised of a variety of workflows that diffuse the responsibility for data quality between stakeholders.

**Table 1.** Participant reported challenges, accommodations, and recommendations for electronic health record data bias reduction.

Perspectives	Challenges	Accommodations and recommendations
Data collection by HCPs^a^ within clinical settings	SDOH^b^ data documentation is dependent upon HCPs' motivation and comfort Influence of discomfort, distrust, stigma, and discrimination Influence of HCP priorities, interpretations, and biases Variance in SDOH data documentation impacts the completeness of EHR^c^ data No incentives for HCPs for recording nonbillable information (eg, SDOH) EHR systems are designed for clinical care provision and billing, which limits SDOH input fields and a loss of potential data that may capture health disparities EHR data accuracy and completeness is not reviewed	Increase patient access to their EHRs Provide patients the ability to add and edit the SDOH information within their EHRs Request HCP feedback on EHR systems Implement patient satisfaction surveys
Data curation: by data scientists	Missing information, mislabeled data, and deidentification practices lead to incomplete datasets that are characterized by underrepresentation Small group records must be suppressed or deleted to protect the privacy of people living with HIV Incomplete data may be requested by clients to be removed Based on available data, SDOH analyses are limited to only race and ethnicity	Standardize and document data curation processes to avoid errors Respond to inquiries regarding missing data Educate clients and researchers on data limitations and responsible data use Guide and verify client and researcher data interpretation
Data management and usage: by public health professionals	Delayed data submission (eg, late laboratory findings) results in inaccurate surveillance records Incomplete data sent from clinics compromises the completeness of the data Inconsistent reporting of SDOH data contributes to the underrepresentation of minority populations Analyses relying on incomplete and inconsistent data risk not capturing minority populations’ experiences	Systematically clean data Statistically compensate for issues with data completion and error and for challenges to data quality Understand data limitations Cross-match data across the state Request customization to data collection systems to better reflect local context and needs Educate the community on department use of data and engage in community stigma management Prioritize preventing opportunities for bias in data application

a HCP: health care provider.

b SDOH: social determinants of health.

c EHR:electronic health record.

### Data Collection by HCPs Within Clinical Settings

At medical appointments, the recording of SDOH information of people living with HIV was dependent on a variety of factors: (1) HCP motivation and comfort to inquire about SDOH information, and (2) people living with HIV’s comfort and trust in their health care provider. Both HCP participants and people living with HIV described comfort in discussing sensitive information in HIV clinical settings. Comfort was identified as stemming from the training HCPs receive to build patient rapport. For example, HCP 1 describes:

*Again, I can’t speak to other offices or other clinics who do the same thing that we do [at the HIV clinic]. But a lot of times with our providers, they’re most often extremely sensitive to the social thing. And so oftentimes, we run behind on time, right? Because providers have a certain amount of time that they are allotted to spend with a patient. And it’s almost never that short. It’s always grows beyond that, and that a lot of times is hinged on the social stuff on these conversations that the patients will have that a little bit beyond just you taking your medication? Cool. How do you feel here? Let me check this. Let’s get labs, right.*

[HCP 1]

Comfort discussing SDOH information in clinical settings is in contrast with non-HIV clinical settings, where HCP participants and people living with HIV described discomfort and HCP stigma. Further, HCPs describe the disincentive, by health care systems, to inquire and record SDOH information into EHRs because, as it is nonbillable information, it is not rewarded. HCPs described experiencing variability in what, when, and how their colleagues documented SDOH information of people living with HIV.

*But, you know, like, I don’t put in anyone’s address. I don’t put anyone’s phone number. I don’t put anyone’s ethnicity. I don’t put in anyone’s gender. I don’t, I know that you can put in that they’re transgender, you could put in their preferred name. Sometimes they’ll have a full name change. Sometimes it’ll be put in as a nickname. Sometimes you’ll have their dead name and then their preferred name as in quotations after too, so it depends on how it’s done and who does it.*

[HCP 2]

Relatedly, HCPs describe a lack of a standard field to enter information that is inclusive of sexual and gender minorities (eg, transgender, nonbinary, and preferred/lived name vs dead name). The SDOH information recorded into EHRs varies by HCP and their perception and interpretation of what is relevant. For example, one person living with HIV noted:

*So, when you meet an older physician, if they get surprised when they finally look at you and they see like oh, okay, okay, well, this is what you are assigned at birth. So this is how I’m going to speak to you. It doesn’t matter. [Good]-bye your pronouns, this is what you’re assigned at birth, so this is what I’m gonna refer to you as. I don’t know how to deal with that, I don’t know how to speak to you. And this is just what I’m used to, this is how I was trained, so this is what it’s going to be.*

[ person living with HIV]

HCPs identified checking charts (eg, review for notes, further information, and compliance), in the clinical setting, as an opportunity to reduce EHR data bias. One challenge to EHR data quality is that the review of patient records, for quality assurance, falls beyond the purview of a specific employee’s role. In order to address EHRs of varying quality, HCPs described the need for EHR review to be included within their employment responsibilities, to have their administrative responsibilities acknowledged, and to be properly compensated.

*Should is a funny word. I think that there should be committed individuals who do clinical care, who have a portion of their time paid for to commit to [checking the quality of patient records]. And I think that departments should have a champion for this kind of work. And working and navigating the medical record in general. Just like in our division, we have champions for antibiotic stewardship, and infection prevention about like spreading diseases around the hospital, and all these other things, you know, we wear those hats, those are recognized as part of the work that we do administratively for the hospital.*

[HCP 4]

People living with HIV and HCPs both recommended patient access to EHR records, through a patient portal, as an opportunity to minimize EHR data bias. Providing people living with HIV with access to their EHRs was viewed as a way in which they could update their SDOH and identity information (ie, preferred name, gender identity, and sexual orientation). In addition, people living with HIV recommended that care experience surveys be used to help capture experiences of bias during their medical appointments. Furthermore, HCPs expressed their desire to provide feedback on their experience using EHR systems, as one participant mentioned, “No one’s asked me” (HCP 1).

### Data Curation by Data Scientists

Data scientists’ workflows within the EHR data lifecycle revolve around their responsibilities in data cleaning, curation, and collaboration with researchers. A primary challenge expressed by data scientists is the challenge of under-representative data that occurs as a result of missing SDOH information (eg, race, ethnicity, and sexual orientation) and mislabeled information (eg, gender identity), within EHRs. When asked why this challenge persists, data scientists explained that it is a result of EHR systems being designed for medical billing, as opposed to research, and the limitation of EHRs to capture only those engaged in care. A data scientist described the potential to address the limitations of EHRs, expanding the utility beyond billing, when they said:

*[…] Nobody’s looking at completeness of recording those data elements or, or accuracy or completeness or. But I do think some of the EHRs can collect that type of thing if the hospital or healthcare provider or organization that owns the data mandates that, you know, or says that that is important, and you need to record it on every patient or every client.*

[Data scientist 1]

One HCP detailed the limitations of EHR data within the research setting, based on their experience, when they noted:

*So the net that we’re getting to actually put things in the medical record is very limited by who’s actually showing up to be seen, because a lot of people don’t trust healthcare providers, or the overall system for that reason. […] I think the methods for [using EHR data] has a lot of shortcomings. Like a lot of times, [researchers and data scientists] will pull a population based on a billing code, and doctors do not always bill to match what’s clinically significant with the patient… and a lot of documentation has discrepancies between what’s clinically significant to the doctors and the way things pass through bureaucracy for processing of that person as entity within a larger financial system. So, like cause of death often does not match the cause of death, as seen by the physician. It’s usually much more vague or completely misleading, and the billing codes are often far, far short of what’s actually happening in the patient, clinically, or missed altogether.*

[HCP 4]

Data scientists describe coping with these challenges, within their workflows, beginning with the raw data received from hospital systems (ie, both HIV-specific and non-HIV-specific). The raw EHR data are first encrypted, deidentified, and then cleaned. A documented protocol is followed during data cleaning to avoid opportunities for errors, through which the data is checked for any general issues and missingness. One data scientist describes the role of documentation and unique identifiers when they share:

*How we avoid errors and that we do have a document that describes our process, generally, of how that that unique identification number is assigned, that uses a combination of data elements, such as first name, last name, social security number and date of birth, but it also can use things like race and sex.*

[Data scientist 1]

Once the EHR data are cleaned, data scientists’ responsibilities shift to ensuring the appropriate use and application of the data by researchers. The data scientists act as data stewards and aim to minimize opportunities for bias by limiting the type and amount of data researchers have access to as well as by providing oversight to ensure that the data that is requested is appropriate for its intended use. The role of data scientists as data stewards was described when a participant shared,

*But as far as the research process, a researcher will usually kind of contact us, [name redacted] and I usually will kind of be, you know, between the two of us will either both of us or one of us will be the ones that kind of initiate the or have those initial conversations, to discuss kind of, you know, what are they studying? And what information do we have available that could help answer that and, and, you know, once we kind of establish that, because we are neutral, we are not data owners, we are data stewards […].*

[Data scientist 3]

Data scientists highlighted their efforts to educate researchers on the limitations of their requested data, responsible data use, statistical analysis, and data interpretation of requested datasets. For example, data scientists educate researchers on how to use external population-level datasets to gauge the representativeness of their data.

### Data Management and Usage by Public Health Professionals

Within the EHR data lifecycle, health department professionals have two key workflow processes based on their responsibilities for HIV surveillance and retention-in-care. HIV surveillance professionals describe challenges in receiving EHR data that is improperly collected or incomplete. Similar to the data scientists above, underrepresentation in EHR data, as a result of SDOH information being inconsistently recorded, persists as a challenge among health department professionals working in HIV surveillance. A focus group of health department professionals describes:

*[…]so there’s […] this constant miscommunication between the field staff and the like in our [name of health department] county clinics. Sometimes they don’t collect the data the right way, and it comes here and the [HIV surveillance] staff have to interpret some way to put it in. So there’s a lot of noise between the point of collection and the point of analysis.*

[Health department professionals focus group 1]

Moreover, HIV surveillance professionals noted that because their system is part of a larger federal reporting system, the input fields are not customizable to capture local needs and do not allow interoperability with other EHR systems. Therefore, the needs of minority populations are not representatively captured. HIV surveillance specialists overcome these data limitations by using appropriate statistical techniques to complete incomplete EHRs.

Retention-in-care specialists rely on EHR data to assess which individuals are retained in HIV care and which require outreach to encourage them to become engaged in care. Among retention-in-care specialists, a primary challenge faced is the late submission of laboratory results. Delayed laboratory results require retention-in-care specialists to assume that an individual is not engaged in care, which requires a follow-up via phone call to encourage them to return to HIV care. For example, a health department professional explained:

*The inability to receive timely labs [is the biggest challenge]. If the physicians are not sending in their labs on time, and there’s a number of non-electronic reports that are coming in, so providers are still using the phone or paper trail to let surveillance know that a person is HIV positive and here are their labs; And they’re not automatically sending them in where it goes right into the system. [Lab records] are having to be manually entered. And with that, because we [at name of engagement in care program] use surveillance systems to do our work and identify individuals, what happens is that sometimes a person may have gone to the doctor and have gotten updated labs… But based on our records those labs haven’t been entered, or they’re somewhere in a repository. And they, it causes us to call a person and say, hey, you’ve been identified as someone who is not in in medical care for a certain [mandatory reportable] condition. […]. So again, that puts us in a bad situation, and it causes heightened tension for the [person living with HIV], because they, are in their mind doing the right thing… But our records are not up to date.*

[Health department professional 1]

In an attempt to overcome the challenges stemming from delayed laboratory results, retention-in-care specialists reported checking with disease surveillance professionals to first see if they were submitted using nonelectronic methods (eg, paper and phone). If not electrically reported, then laboratory results are manually entered and reviewed across departments to accurately reflect those who are and are not retained in care. Further, before directly contacting an individual, retention-in-care specialists reported cross-matching patient records with other databases (eg, government-funded HIV clinics, state and federal justice systems, and obituaries). One health department professional described this process when they shared:

*We’re able to conduct record searches with other states to confirm if they have or have a person that mimics our demographics here in South Carolina. So, we can see if that person is in their state, and if they’re receiving care that way. We check obituaries, and we do a match with vital records to identify individuals who have been deceased. And then some people kind of link on their own, or there’re variety of ways that we go about ensuring that a person is truly not in care in South Carolina before we reach out to them…. because we don’t want to heighten their stigma, we want them to feel comfortable. We don’t want them to have some random person [contacting them] because they don’t know the staff.*

[Health department professional 1]

Health department professionals described community apprehension and fear of HIV surveillance and retention-in-care efforts due to a lack of transparency and uncertainty as to how the state and federal governments would use their personal information. Community outreach efforts have been initiated in efforts to assuage community members’ fears and increase transparency surrounding these data collection methods and uses.

## Discussion

### Summary of Key Findings

The present study explored the workflow processes of key stakeholders across the EHR data lifecycle, including HIV clinics, data scientists, and public health departments. Instances where bias is introduced to EHR data were found throughout the stages of data collection, curation, management, and usage. Within the data collection workflow, HCP implicit biases and discomfort soliciting SDOH information of people living with HIV, variation in HCP SDOH information recording, and lack of standardized EHR fields and protocols introduced bias into the resulting EHR data. Within data curation, management, and usage workflows, challenges that introduced bias included receiving poor quality data (eg, inaccurate and incomplete) that is not standardized and is submitted in an untimely manner. Our findings contribute an important perspective to the literature as they illustrate EHR data’s vulnerability to social biases (eg, structural, institutional, and interpersonal), and the subsequent vulnerability of the associated research products to reproduce and exacerbate these biases. These implications are particularly relevant in an era where EHR data are frequently used as a data source in Big Data and Artificial Intelligence research [30,31].

### Reducing Opportunities for Bias at Data Collection via Patient Portals and Patient-Experience Surveys

Within the clinical setting, HCPs input patient information into EHR systems, a process that is vulnerable to introducing bias that may compromise EHR data quality (eg, accuracy and completeness). A systematic review of the literature described the prevalence of implicit biases amongst HCPs and their association with a decrease in the quality of care received; within the present study, these implicit biases were identified to impact the patient information collected and reported [32]. Biases impacting EHR data are introduced when SDOH data is systematically missing or under-reported, thereby omitting or erasing some populations from the literature [33]. For example, studies conducted at a variety of academic medical centers found stigmatizing language within EHRs and missing or discrepant SDOH information (ie, racial, ethnic, and gender identity) between HCP and patient reports [34-37]. People living with HIV and HCPs, within this study, recommended increasing patient access to EHRs, via patient portals, to increase the accuracy and completeness of patient information through self-report, which has been referred to as the gold standard in the literature [33,36,37].

The existing literature notes improved accuracy and completeness of records when patients are given access to their EHRs [20,38]. From the HCP perspective, literature has found a positive attitude among chronic disease providers (eg, oncologists), where open access to EHRs is perceived as cultivating a welcoming care environment with improved patient-provider communication [39]. Additional benefits of patient EHR access have been identified within the literature to include increased engagement, knowledge, and transparency, as well as reduced anxiety, improved patient-provider relationships, and improved health outcomes [40]. Notably, patients with chronic conditions that require continual care (eg, HIV) or experience emergent health events (eg, new HIV diagnosis) have been identified as increasingly likely to benefit from EHR access (eg, portal-based laboratory results) [40]. Despite the potential benefits of patient portals, they must also be weighed against the possible barriers and negative effects, such as inaccessibility, privacy and confidentiality concerns, health and tech literacy limitations, and the exclusion of vulnerable populations [40-44]. Further research is needed to elucidate the benefits, barriers, and effects of EHR amendment through patient portals in the HIV clinical setting to ensure the accuracy and completeness of EHR data is equitable among people living with HIV belonging to all backgrounds, and to avoid further exacerbating health disparities.

In addition to the recommendation to increase the accuracy and completeness of SDOH information through patient portals, participants also suggested the implementation of patient-experience surveys to mitigate potential biases during EHR data collection. Within the literature, positive patient experiences have been associated with better clinical outcomes and patient safety, thereby demonstrating the utility and importance of rigorous (ie, tailored, validated, sufficiently powered, standardized protocols, and goal-oriented) patient-experience surveys [45,46]. Where patient-experience surveys are traditionally used to evaluate health care quality within health care systems, within the present context, the surveys offer an opportunity for people living with HIV to document their experiences and provide feedback that is essential to identifying and understanding the potentially differing experiences of patients [47-49]. For example, patient-experience surveys could timely and responsively elucidate patterns in the care environment, such as where there is a discrepancy between HCP hesitation to inquire about patients’ sexual orientation or gender identity and patients’ willingness to disclose due to the perceived benefits of individualized care resulting from their disclosure [50]. When developed and implemented according to best practices, patient-experience surveys could be a viable tool to identify and address interpersonal, institutional, and structural biases occurring within HIV-specific care settings, subsequently warranting future research.

As concerns about EHR data quality begin at data collection, improved EHR accuracy and completeness could preliminarily address emergent concerns and limitations. Interventions addressing EHR data collection should consider the effect of shifting the responsibility from providers to patients, how to mitigate the effect of HCPs’ implicit biases, HCP perceptions of EHR, and HCP EHR burnout, among others [38,40,51,52]. Due to the facilitators and challenges to EHR data collection, completeness, and accuracy in the clinical setting, further research is needed to evaluate the applicability and feasibility of patient and HCP-recommended actions (ie, patient portals and patient-experience surveys) to cope with challenges to EHR data quality. Further research in this domain is urgently called for due to the potentially harmful effects of drawing conclusions, in health disparities research, based upon data of which the quality is not guaranteed (eg, inaccuracy and missingness), as it risks perpetuating structural biases and systems of oppression inherent in EHR data [36,38,53,54].

### Reducing Underrepresentation in EHR Data Through Improving Access and Engagement in Care

Data scientists and health department professionals primarily described data delays, missingness, and underrepresentation as challenges faced when using EHR data to draw statistical conclusions and develop public health surveillance–based decision-making tools. In efforts to cope with the susceptibility of EHR data to bias, the existing literature offers statistical techniques (eg, multiple imputation and likelihood-based estimation inverse probability weighting) and the use of external datasets to assess the representativeness of EHR data [17,18,20]. The limitations of representativeness identified here diverge from previous literature that did not identify representativeness as a possible source of error within the EHR data lifecycle [55]. Within a deep south context, underrepresentation may be best proactively addressed through increasing the affordability, availability, and accessibility of care for populations not engaged in care [56]. Targeting determinants and barriers to care would address disparities experienced by racial minority or sexual minority populations who face an increased likelihood of the inability, or a delayed ability, to access health care services due to not being able to afford health care services and medication [57,58]. As such, we recommend that further research is needed to structurally address the SDOH and barriers to care and to innovatively engage stigmatized and marginalized populations in the Deep South that have been historically excluded from HIV care (eg, undocumented individuals, young men who have sex with men, and individuals engaging in illicit substance use).

The generalizability of EHR-derived results to the broader population may be complicated because patients represented in EHR systems are more likely to be ill, leading to informed presence bias [20,59,60]. However, informed presence bias may differ in the HIV context because HIV is a mandatorily reportable condition in the United States (except in Idaho and the US Virgin Islands), where diagnoses and progress toward management must be reported by all health care practitioners, to state health departments, regardless of an individual’s health status [61]. Therefore, HIV studies using EHR data may be more generalizable than other disease contexts because informed presence bias (ie, a form of selection bias) may not be operating within HIV-specific EHR datasets to the same extent that it would within other chronic disease contexts. Despite these considerations, there persist biases and limitations to HIV-specific EHR data, such as delayed linkage to care, omission of vulnerable populations living with HIV (eg, unhoused populations and people who use illicit substances), and barriers to care (eg, stigma and lack of health insurance) [62]. As such, further research is needed to evaluate the generalizability and representation of vulnerable populations living with HIV within HIV-specific EHR data at the state, regional, and national levels. Although statistical techniques can be used to cope with challenges to representativeness, there is a demonstrated need to decrease barriers to care and to engage all people living with HIV in care, thereby addressing missingness within the clinical setting.

### Increasing Opportunities for Stakeholder Collaboration to Reduce Opportunities for EHR Data Bias

Data scientists and health department professionals act as stakeholders in the EHR data lifecycle due to their responsibilities in data curation, management, and usage. In the present study, common challenges to EHR data curation and usage, that are instances in which bias may be introduced, include untimely data submission, nonuniform data submission, and the submission of inaccurate and incomplete records. In efforts to cope with these challenges and subsequent negative effects, health department professionals described their need to communicate with other key stakeholders in the EHR data lifecycle. For example, health department professionals see a need to discuss knowledge of and standards for reporting HIV diagnoses and progress toward viral suppression (eg, timeliness, reporting format, completeness of data, and types of information requested), with HCPs. Within the present HIV-specific context, further research is needed to evaluate how HIV reporting standard adherence may differ between Ryan White and private HCPs, as the former were anecdotally perceived as being more adherent to HIV reporting standards and differential adherence to reporting standards could result in biased EHR data. Further, due to the number of stakeholders responsible for data collection, curation, management, and usage, there is an observed diffusion of responsibility where data quality persists as an ongoing issue and the ability to enact change is perceived as limited because cross-facility and cross-department protocols fall outside the scope of stakeholders’ roles. Efforts to improve the quality of EHR data must strengthen the collaboration between key stakeholders across the EHR data lifecycle (eg, HCPs, data scientists, and health department professionals) [17,63].

### Methodological Considerations

This study has both strengths and limitations. In regard to study strengths, transferability was increased by providing thick descriptions of participant perspectives via transcript quotations [64]. As a result of our study design, our study results may be transferable to other settings in the Southeastern United States that resemble South Carolina’s HIV disease burden, infectious disease reporting practices, and health system structure [65]. Moreover, our study results may also be applicable to similar settings in the Southeast that use EHR systems with limited integration and have limited practices in place to address social and statistical biases. Furthermore, confirmability was increased through the use of a reflexive journal and an analysis matrix [64]. Credibility was increased through the use of an audit trail [64]. Finally, confirmability, credibility, and dependability were increased through triangulation by collecting data from multiple perspectives (ie, people living with HIV, HCPs, data scientists, and health department professionals) [64]. This study is also characterized by a few limitations. Namely, a limitation of the present study is that saturation was not reached, based on the researchers’ perspectives that additional interviews could have yielded additional findings [23]. The ability to reach saturation was limited by time and resource constraints as well as participant accessibility and availability. As a result, further interviews could have provided additional experiences from stakeholders (eg, public health researchers) not captured within the present study. The inability to capture the perspectives and experiences of stakeholders who use EHR data omits the subsequent challenges and needs of those who rely upon the data to create the knowledge base, thereby necessitating further research of a larger sample size that includes such perspectives. Transferability may be limited to only a portion of the EHR data lifecycle (ie, collection, curation, and management) as the full breadth of stakeholders was not included in the present study. Finally, since saturation was not reached within the present study, future qualitative and quantitative studies should be conducted to explore the dimensions of the phenomenon further.

### Conclusions

Regardless of how data collection, curation, management, and usage workflow processes were structured, challenges and pitfalls across the EHR data lifecycle created opportunities for bias to be introduced. The number of times EHR data changes hands over its lifecycle (eg, various HCPs collect and enter patient information, data scientists and health department professionals clean and analyze data, researchers and public health professionals use data) and the limited oversight between stakeholders were identified as two primary avenues through which bias is introduced into EHR data. Based upon the existing literature and perspectives of the stakeholders represented in the present findings, recommendations for action to reduce bias in EHR data include both structural intervention (eg, SDOH, health care access and quality, and health equity) and EHR-specific recommendations to expand EHR data collection beyond what is necessary for billing, due to its increased use within research; to seek and operationalize the input of key stakeholders regarding what information is included and required in EHR systems; to facilitate stakeholder collaboration (eg, develop a shared data cleaning protocol to avoid duplicative efforts); and to conduct trainings for HCPs regarding the impact of HCP implicit biases on patient experiences and health outcomes, the best practices for EHR data recording, and how EHR data is used within research and decision-making. Within a larger public health context, these findings and recommendations serve to inform health care systems, public health policymakers, and stakeholders on the potential biases, benefits, and challenges to using EHR data in their research, in the interest of improving EHR data quality.

## Supplementary material

10.2196/71388 Checklist 1 Standards for Reporting Qualitative Research (SRQR) checklist.

## Abbreviations

EHR: electronic health records
GDPR: General Data Protection Regulation
HCP: health care provider
LGBTQ: lesbian, gay, bisexual, transgender, and queer
NIH: National Institutes of Health
SDOH: Social Determinants of Health
SRQR: Standards for Reporting Qualitative Research
